# Back to ED basics - GP and health practitioner eating disorders education program

**DOI:** 10.1186/2050-2974-3-S1-O15

**Published:** 2015-11-23

**Authors:** Jennifer Beveridge, Karen Philippzig, Michelle Roberton

**Affiliations:** 1 Eating Disorders Victoria, Australia; 2 Centre for Excellence in Eating Disorders, Australia

## 

Eating Disorders Victoria (EDV) has been providing information and support to people affected by eating disorders for over 30 years. Feedback has been received regularly from callers to the helpline, participants in support groups and members regarding General Practitioners' (GP) quality of knowledge about eating disorders and sensitivity to issues around disclosure. In 2013, EDV obtained 3 years funding from the William Buckland Foundation to provide an eating disorders education program for primary healthcare professionals.

The program, which will be described in more detail, consists of three components:

1. Information brochures in over 880 clinics throughout Victoria in partnership with distributors, InfoMed

2. RACGP accredited ‘Eating Disorders in General Practice’ education sessions, developed and provided in partnership with the Victorian Centre for Excellence in Eating Disorders (CEED)

3. Visits to Victorian GP clinics to provide opportunities for informal and individualised information and support to staff

Preliminary outcomes have been positive, with more than 70% of the 430 participants reporting that their learning needs regarding their ability to recognise the signs & symptoms of the different types of eating disorders were entirely met. Other positive outcomes will be presented and implications for future work will be discussed.

